# IS*26* Family Members IS*257* and IS*1216* Also Form Cointegrates by Copy-In and Targeted Conservative Routes

**DOI:** 10.1128/mSphere.00811-19

**Published:** 2020-01-08

**Authors:** Christopher J. Harmer, Ruth M. Hall

**Affiliations:** a School of Life and Environmental Sciences, The University of Sydney, Sydney, New South Wales, Australia; University of Nebraska Medical Center

**Keywords:** IS*1216*, IS*257*, IS*26*, antibiotic resistance, insertion sequence, mobile genetic element

## Abstract

IS*26* has been shown to form cointegrates both by a copy-in mechanism involving one insertion sequence (IS) and a target and by a targeted conservative mechanism involving two ISs. IS*26* is the flagship of a group of 65 bacterial ISs in the recently redefined IS*6*/IS*26* family. Here, whether other family members can also use two mechanisms was examined using members of the IS*257*/IS*431* and IS*1216* isoform groups, which are associated with antibiotic resistance genes in staphylococci and enterococci, respectively. Transposases Tnp257 and Tnp1216 have 39% and 47% amino acid identities, respectively, with Tnp26 and are 62% identical to one another. Using a novel transposition assay, pUC-based plasmids carrying these ISs integrated into the chromosome of a temperature-sensitive *polA* Escherichia coli strain grown at the restrictive temperature. In the cointegrates, the plasmid carrying IS*257* was flanked by various 8-bp target site duplications, consistent with random target selection. However, in a mating-out assay, only the targeted conservative reaction was detectable at a low frequency in a *recA*-negative E. coli strain, indicating that IS*257* is at least 100-fold less active than IS*26*. For IS*1216*, in mating-out assays, both copy-in and targeted conservative cointegrate formation were detectable at frequencies similar to those observed for IS*26*. Duplication of various 8-bp target sites was detected for the copy-in route. For both IS*257* and IS*1216*, when both of the plasmids carried an IS, the targeted conservative route occurred at a significantly higher frequency than the copy-in route, and only cointegrates formed by the conservative route were detected.

**IMPORTANCE** IS*26* differs from other studied ISs in the reactions that it can undertake. The differences make IS*26* uniquely suited to its key role in the recruitment and spread of antibiotic resistance genes in Gram-negative bacteria. However, whether other ISs in the IS*6*/IS*26* family can perform the same reactions is not known. IS*257*/IS*431* and IS*1216* isoforms found associated with antibiotic resistance genes in the Gram-positive bacteria staphylococci, enterococci, streptococci, and clostridia are related to IS*26*. However, the way that they move had not been investigated, limiting interpretation of their role in resistance gene dissemination and in the formation of cointegrates and complex resistance regions in staphylococci and enterococci. Here, they are shown to share the broad catalytic capabilities of IS*26*, demonstrating that it is likely that all members of the redefined IS*6*/IS*26* family of bacterial ISs likewise are able to use both the copy-in and conservative routes.

## Abstract (precis)

IS*26* differs from other studied ISs in the reactions that it can undertake. The differences make IS*26* uniquely suited to its key role in the recruitment and spread of antibiotic resistance genes in Gram-negative bacteria. However, whether other ISs in the IS*6*/IS*26* family can perform the same reactions is not known. IS*257*/IS*431* and IS*1216* isoforms found associated with antibiotic resistance genes in the Gram-positive bacteria staphylococci, enterococci, streptococci, and clostridia are related to IS*26*. However, the way that they move had not been investigated, limiting interpretation of their role in resistance gene dissemination and in the formation of cointegrates and complex resistance regions in staphylococci and enterococci. Here, they are shown to share the broad catalytic capabilities of IS*26*, demonstrating that it is likely that all members of the redefined IS*6*/IS*26* family of bacterial ISs likewise are able to use both the copy-in and conservative routes.

## INTRODUCTION

The IS*6*/IS*26* family, hereinafter the IS*26* family, includes the insertion sequences (ISs) that are most commonly found associated with antibiotic-resistance genes in resistant Gram-negative (IS*26*) and Gram-positive (IS*257*/IS*431* and IS*1216*) bacteria (1). However, despite their importance, only a few members of the family have been examined experimentally, and only IS*26* has been studied in detail.

**FIG 1:**
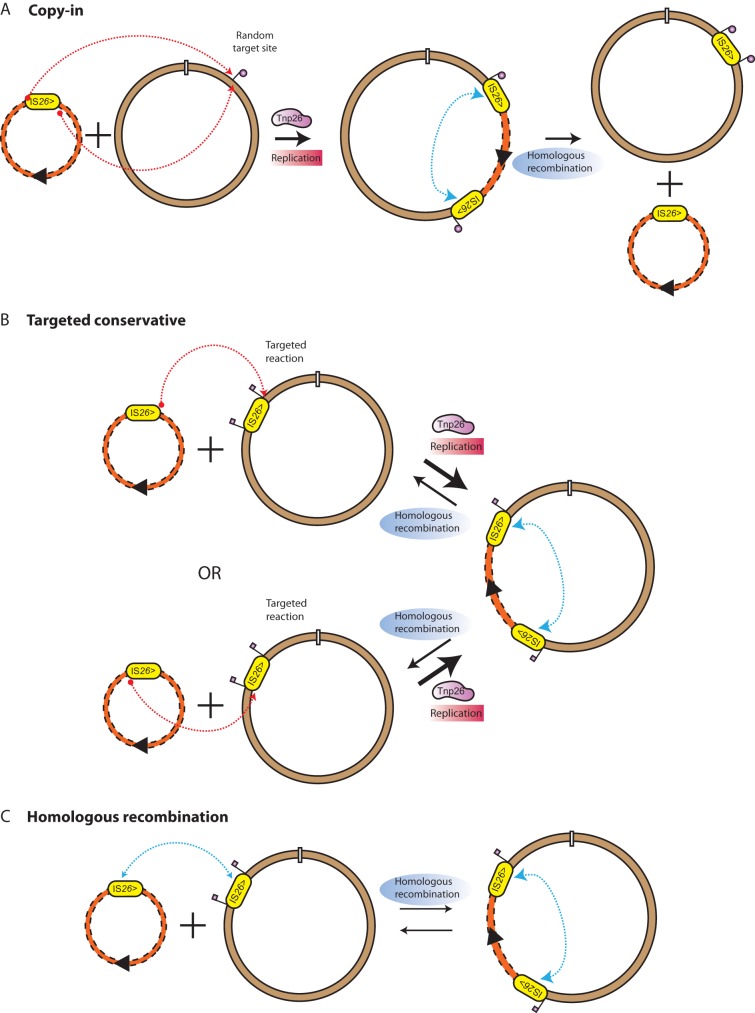
Three routes to cointegrate formation between two molecules. (A) Copy-in route; (B) targeted conservative route; (C) homologous recombination. IS*26* is indicated by yellow ovals, with orientation shown by “>.” A target site and subsequent 8-bp duplications are indicated by a vertical flag. The relative frequencies of the Tnp26-mediated and homologous-recombination-mediated reactions are indicated by the thicknesses of the arrows.

IS*26* movement was first examined in the 1980s. Rather than moving alone to a new location, IS*26* was shown to use a characteristic “replicative” mechanism to exclusively form cointegrates between two DNA molecules, a donor molecule containing an IS*26* and a target molecule (2–4). This route duplicates the IS and the 8-bp target site (Fig. 1A). Apparent simple transposition products, namely, a single IS*26* sequence surrounded by an 8-bp target site duplication (TSD) at a random site, likely arise via a cointegrate intermediate. Resolution of the cointegrate then occurs via homologous recombination between the two directly oriented IS copies in the cointegrate (Fig. 1A). The “replicative” route was later renamed “copy-in” to distinguish it from the “copy-out paste-in” mechanism used by other ISs, which was discovered subsequently and also involves a replicative step (5).

For 3 decades, copy-in cointegration was believed to be the sole movement mechanism used by IS*26* and related ISs. However, IS*26* was recently predicted, and then shown, to utilize a second transposase-dependent reaction to form cointegrates when both of the two DNA molecules involved carry a copy of IS*26* (6). This unique reaction differs from any described for any other ISs to date and has properties akin to site-specific recombination. The reaction is targeted and occurs at one or the other end of the two ISs (7). It is also conservative, as the IS is not duplicated and a TSD is not generated (Fig. 1B) (6). Cointegration via the conservative route occurs at a frequency over 50-fold higher than with copy-in cointegrate formation (6–8), making it the preferred reaction if copies of IS*26* in two different DNA molecules are available. Though the same cointegrate may be formed by homologous recombination (Fig. 1C), the transposase-catalyzed reaction has been shown to occur at a frequency over 1,000-fold higher than that of homologous recombination (8), making it the preferred reaction in a recombination-proficient host.

Recently, the relationships within a curated set of 112 ISs currently assigned to the IS*6* family in ISfinder (https://isfinder.biotoul.fr/) were examined to identify the ISs that are the closest relatives of IS*26* and hence most likely to also utilize the targeted conservative cointegrate formation mechanism discovered for IS*26* (9). A well-differentiated group of 65 bacterial ISs was defined as the IS*6*/IS*26* family to distinguish it from the larger IS*6* family documented in ISfinder (9). The reduced family, here referred to as the IS*26* family, includes six clades (clades I to VI), with IS*26* belonging to clade I and members of the IS*257*/IS*431* and IS*1216* isoform groups belonging to clade II, which includes most of the ISs of Gram-positive origin (9).

Members of the IS*257/*IS*431* group are found in *Staphylococcus* species. Three variants named IS*257* (IS*257*R1, IS*257*R2, and IS*257*L) (10, 11) and three variants named IS*431* (IS*431*L, IS*431*R, and IS*431mec*) (12) were discovered contemporaneously, and both names have been used over the years to refer to identical or closely related ISs. Here, we use IS*257*. IS*257* isoforms range in size from 788 to 791 bp, with 18- or 20-bp terminal inverted repeats (TIRs), and share >95% nucleotide identity. The 224-amino-acid transposases share >98% amino acid identity with one another (see reference 9 for details of the variation).

Studies of available DNA sequences have inferred the ability of IS*257* to form cointegrates and indicated an 8-bp TSD (13, 14). In an early study, IS*257*R2 was shown to be active. Cointegrates were formed between two plasmids via the untargeted copy-in route when only one plasmid contained a copy of IS*257*. The IS*257* was duplicated and an 8-bp TSD created (15). However, the frequency of cointegrate formation was not recorded. In the same study, cointegrates were formed between two plasmids, each containing a copy of IS*257*, and the cointegrates appeared to have formed by recombination between the two IS*257* sequences (15). As this experiment was conducted in a recombination-proficient Staphylococcus aureus strain, homologous recombination could not be excluded as the mechanism responsible, though it was claimed that the frequency of cointegration was higher than expected if homologous recombination was solely responsible (15).

IS*1216* isoforms are 809 bp, with perfect 19-bp TIRs. A single study has inferred the importance of IS*1216*-mediated formation of cointegrate plasmids for mobilizing resistance genes from Enterococcus faecium to Enterococcus faecalis and reported the presence of an 8-bp TSD in the cointegrate (16). However, the frequency of cointegrate formation has never been quantified, and the possibility that IS*1216* utilizes the targeted conservative mechanism has not been considered or examined.

**FIG 2:**
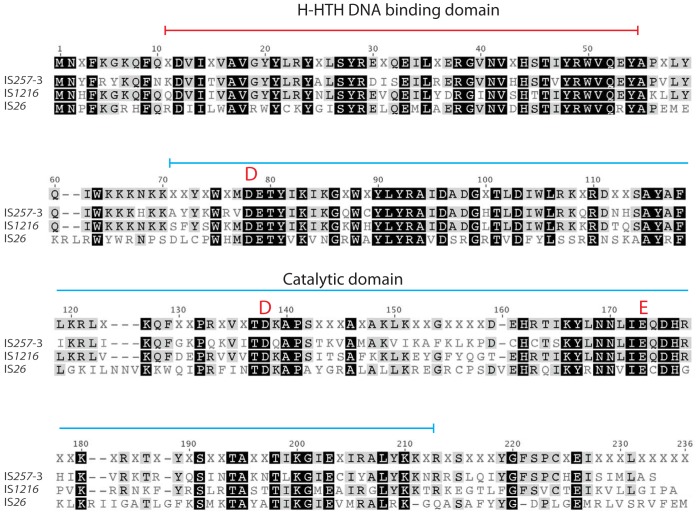
Alignment of the amino acid sequences of the transposases of IS*26*, IS*257*-3, and IS*1216*. The extents of the H-HTH putative DNA binding domain and the DDE catalytic domain are marked above the sequences. The completely conserved DDE residues are marked by red letters. Amino acids are indicated as follows: black background, 100% similarity; dark-gray background, 80 to 99% similarity; unshaded letters, less than 79% similarity.

Here, to determine whether the targeted conservative cointegrate formation mechanism is unique to IS*26* or whether other distantly related ISs in the IS*26* family can also use this mechanism, IS*257* and IS*1216* were examined. The transposases of IS*26*, IS*1216*, and IS*257* share between 39 and 62% amino acid identity with each other (Table 1), and an alignment of the transposases (Fig. 2) shows high conservation throughout the helix helix-turn-helix (H-HTH) DNA binding domain and the catalytic domain (see Fig. S1 in reference 9 for a full alignment of the IS*26* family). To eliminate the detection of products of homologous recombination, cointegration was experimentally determined in a *recA*-negative Escherichia coli strain using well-established assays for detecting and quantifying cointegrate formation via both the untargeted copy-in route and the targeted conservative route.

**TABLE 1:** Transposase amino acid similarity

IS	% amino acid similarity to transposase of:			
	IS*26*	IS*257*R2	IS*257*-3	IS*1216*
IS*26*	100.0	38.7	39.1	46.7
IS*257*R2		100.0	99.1	61.9
IS*257*-3			100.0	61.8
IS*1216*				100.0

## RESULTS

### IS*257*-mediated copy-in cointegrate formation.

IS*257*-mediated cointegrate formation was initially examined in E. coli using a mating-out assay to detect cointegrates formed between the conjugative plasmid R388 (trimethoprim resistant [Tp^r^]) and pRMH1008 (ampicillin resistant [Ap^r^]), a small, nonconjugative nonmobilizable plasmid containing IS*257*-3. However, cointegrate formation between pRMH1008 (IS*257*-3) and R388 was not detected (see below). To eliminate the possibility that the IS*257*-3 variant chosen was defective, a second variant (IS*257*R2) was cloned from pSK41 to generate plasmid pRMH1009. The transposase of IS*257*R2 differs from the transposase of IS*257*-3 at three positions: 37E→G, 75V→I, and 96D→E. Cointegrate formation was also not detected between pRMH1009 and R388, suggesting that either the reaction occurred at a frequency below the limit of detection of this assay (∼8 × 10^−8^ cointegrates per transconjugant) or that IS*257*, which is found exclusively in *Staphylococcus* spp., may not be active in E. coli.

**FIG 3:**
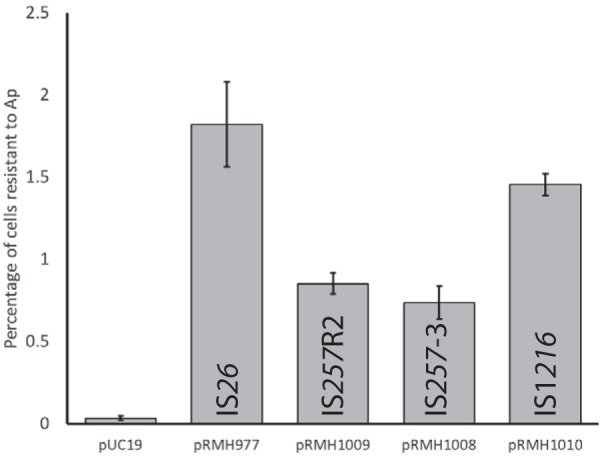
Cointegrate formation between pUC19 derivatives and the MM383 chromosome. The percentage of cells resistant to ampicillin (Ap), indicative of stable cointegrate formation between the plasmid and the MM383 chromosome, is shown as the means of results from three independent experiments. Error bars indicate the standard errors of the means.

In order to determine whether IS*257*-mediated cointegrate formation occurs at a frequency below that detectable by the standard mating-out assay, a *polA* mutant strain, E. coli MM383, which produces a DNA polymerase I (PolI) that is defective at 42°C, was used to detect cointegrate formation as described previously (17). Resistance to Ap (mediated by the *bla*_TEM-1_ gene in the pUC19 backbone) after growth at the nonpermissive temperature is indicative of the incorporation of the plasmid into the chromosome. The IS*26*-containing pUC19 derivative pRMH977, which is known to form cointegrates (6), was first tested to validate the assay. When pUC19 was used as a control, i.e., there was no IS present to mediate cointegrate formation, only 0.03% of cells retained resistance to Ap following 24 h of growth at 42°C without Ap selection (Fig. 3). In contrast, when MM383 containing pRMH977 was grown for 24 h at the nonpermissive temperature without selection, 1.67% (average from three independent experiments) of cells retained Ap resistance (Fig. 3).

To examine IS*257*-mediated cointegrate formation, MM383 containing either pRMH1008 (IS*257*-3) or pRMH1009 (IS*257*R2) was grown for 24 h without selection at the nonpermissive temperature. After being plated on selective media, 0.85% or 0.74% of cells (means from three independent experiments) from the MM383/pRMH1008 or MM383/pRMH1009 culture, respectively, retained Ap resistance (Fig. 3). To determine whether the Ap resistance was due to cointegrate formation between pRMH1008 or pRMH1009 and the MM383 chromosome or was due to residual free plasmid, 30 presumptive cointegrates (15 each from pRMH1008/MM383 and pRMH1009/MM383) were regrown for a second 24-h cycle at 42°C without Ap selection. In all 30 cases, 100% of cells in the culture retained resistance to Ap, confirming that IS*257*-3 and IS*257*R2 had formed stable cointegrates with the MM383 chromosome.

Fifteen cointegrates formed between pRMH1009 (IS*257*R2) and the MM383 chromosome were subjected to inverse PCR and sequenced using outward-facing primers in IS*257*R2 (RH2736 and RH2737) to determine the location of pRMH1009 and to determine whether an 8-bp target site duplication was generated. pRMH1009 was integrated at 15 different locations and in both possible orientations within the MM383 chromosome (Table 2). In each instance, the two copies of IS*257*R2 were in the same orientation and an 8-bp TSD had been generated, as expected for an IS*26* family member.

**TABLE 2:** Locations of cointegrates formed between pRMH1009 (IS*257*R2) and E. coli MM383 chromosome

Cointegrate	TSD location^*a*^	TSD sequence	Orientation^*b*^
1	1056978–1056985	ATGGGGGA	1
2	2168492–2168499	TGCCACTG	1
3	1504489–1504496	CAGTGGGT	2
4	4325421–4325428	GGCAAGAT	1
5	3569123–3569130	ATACGACG	2
6	3421503–3421510	CTAACTGG	2
7	2966437–2966444	ACGGAGAT	2
8	37426–37433	TTGAGTGG	1
9	4412738–4412745	TGCTACTA	2
10	203519–203526	GAAGAACT	1
11	3308058–3308065	TCGGATTT	1
12	549300–549307	TGATCGCA	2
13	1852645–1852652	CAACGACA	2
14	3931278–3931285	CTAAGCAC	1
15	4002153–4002160	CGCCAATG	2

a Location in the E. coli K-12 reference sequence (GenBank accession number U00096.3).

b Orientation 1 is defined as that of the *tnp257* of IS*257*R2, which is in the same orientation as the positive strand of the K-12 chromosome. Orientation 2 is defined as that of the *tnp257* of IS*257*R2, which is in the orientation opposite to that of the positive strand of the K-12 chromosome.

### IS*257*-mediated targeted conservative cointegrate formation.

The ability of IS*257* to perform targeted conservative cointegrate formation was tested in a *recA*-negative background to ensure that all events detected were catalyzed by the transposase. Cointegration was tested using pRMH1008 (IS*257*-3 Ap^r^) and R388::IS*257*-3 Tp^r^ or pRMH1009 (IS*257*R2 Ap^r^) and R388::IS*257*R2 (Tp^r^). IS*257*-3 in pRMH1008 formed Ap^r^ Tp^r^ cointegrates with R388::IS*257*-3 at an average frequency of 5.11 × 10^−6^ cointegrates per transconjugant (Table 3). When pRMH1009 was tested, Ap^r^ Tp^r^ cointegrates were formed at a similar average frequency of 3.59 × 10^−6^ cointegrates per transconjugant (Table 3). These frequencies are 40- to 60-fold lower than the values obtained here (Table 3) and our previously reported values (2.1 × 10^−4^ [6] and 2.9 × 10^−4^ [7]) for the reaction between two wild-type IS*26* sequences under the same conditions.

**TABLE 3:** Cointegrate formation frequencies

IS type and IS	Target	Cointegration frequency^*a*^	
		Range	Mean (SD)
Untargeted replicative			
IS*257*-3	R388	<4.32 × 10^–8^ to <9.45 × 10^–8^	<7.47 × 10^–8^
IS*257*R2	R388	<5.55 × 10^–8^ to <8.99 × 10^–8^	<7.84 × 10^–8^
IS*1216*	R388	9.00 × 10^–8^ to 4.14 × 10^–7^	4.47 × 10^–7^ (3.74 × 10^–7^)
IS*26*	R388	2.1 × 10^–7^ to 7.03 × 10^–7^	5.14 × 10^–7^ (2.66 × 10^–7^)

Targeted conservative			
IS*257*-3	R388::IS*257*-3	3.10 × 10^–6^ to 6.67 × 10^–6^	5.11 × 10^–6^ (1.82 × 10^–6^)
IS*257*R2	R388::IS*257*R2	2.30 × 10^–6^ to 5.91 × 10^–6^	3.59 × 10^–6^ (2.02 × 10^–6^)
IS*1216*	R388::IS*1216*	4.33 × 10^–5^ to 9.38 × 10^–5^	6.99 × 10^–5^ (2.53 × 10^–5^)
IS*26*	R388::IS*26*	3.29 × 10^–4^ to 6.09 × 10^–4^	4.88 × 10^–4^ (1.44 × 10^–4^)

a Frequency was measured as the number of cointegrates per transconjugant. The number of replicates was 3 in every case.

PCR screening of 10 Ap^r^ Tp^r^ cointegrates from each of the three experiments confirmed that in all instances, pRMH1008 or pRMH1009 had been incorporated adjacent to the existing IS in R388::IS*257*-3 or R388::IS*257*R2, respectively. Hence, the IS*257* variants IS*257*-3 and IS*257*R2 are able to perform the targeted conservative cointegrate formation reaction previously described for IS*26*.

### Cointegrate formation mediated by IS*1216*.

**FIG 4:**
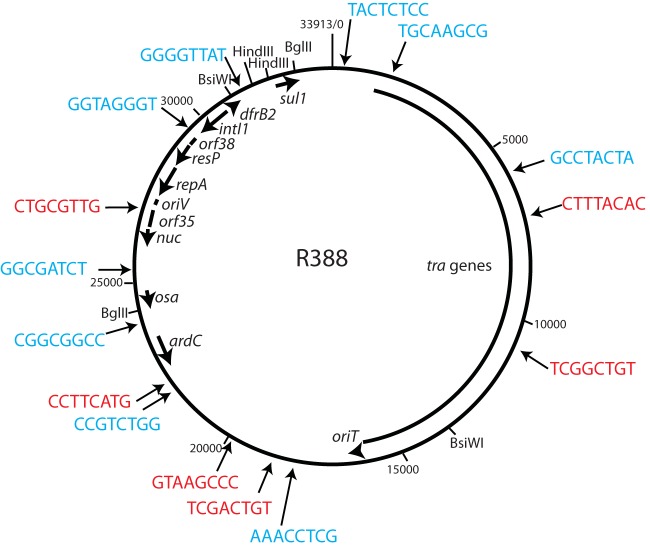
Cointegrate formation between pRMH1010 (IS*1216*) and R388. The R388 backbone is drawn to scale from GenBank accession no. BR000038 with key resistance genes, genes involved in replication (*repA*), and genes involved in conjugative transfer (*tra*) shown as arrows inside the circular backbone. Arrows pointing toward the circular backbone indicate the location of 15 mapped R388::pRMH1010 cointegrates, and the sequence of the 8-bp duplication of the target is shown. Blue lettering indicates that the cointegrate was in orientation 1 (*tnp1216* is in the same orientation as the R388 *repA* gene), and red lettering indicates that the cointegrate was in orientation 2 (*tnp1216* is in the orientation opposite to that of R388 *repA*).

The ability of IS*1216* to perform untargeted copy-in cointegrate formation had never been tested previously. With the standard mating-out assay, cointegrates formed between R388 (Tp^r^) and pRMH1010 (IS*1216* Ap^r^) were detected. The reaction between pRMH1010 and R388 generated Ap^r^ Tp^r^ cointegrates at a frequency of 4.47 × 10^−7^ cointegrates per transconjugant, averaged from three independent experiments (Table 2). This is comparable to the frequency of cointegrate formation demonstrated here (Table 2) and reported previously for IS*26* via this route (7, 18, 19). Fifteen Ap^r^ Tp^r^ cointegrates (five from each of three independent experiments) were subjected to inverse PCR and sequencing to determine the location of the integrated pUC-based plasmid in the R388 backbone. Cointegrates had formed at 15 different positions in R388 (Fig. 4) in both possible orientations. In each instance, IS*1216* had been duplicated, the two copies of IS*1216* were in direct orientation to each other, and an 8-bp TSD was generated.

Untargeted cointegration was also demonstrated using the temperature-sensitive MM383 assay. After 24 h of growth without selection at the nonpermissive temperature, 1.45% of colonies retained Ap resistance (Fig. 3), indicative of cointegrate formation. This is comparable to the frequency of the presence of IS*26* (1.82%) and approximately 2-fold higher than the frequency of the two IS*257* isoforms tested, consistent with the frequency demonstrated in the standard mating-out assay.

Targeted conservative cointegrate formation was also measured using pRMH1010 (Ap^r^) and R388::IS*1216* (Tp^r^). IS*1216* in pRMH1010 formed Ap^r^ Tp^r^ cointegrates with R388::IS*1216* at an average frequency of 6.99 × 10^−5^ cointegrates per transconjugant (Table 2). This frequency is 150-fold higher than the frequency of the untargeted copy-in reaction reported above. It is similar to the frequency obtained for the reaction between two IS*26* sequences here (Table 2) and previously (6, 7, 19). PCR screening of 10 streptomycin-resistant (Sm^r^) and Ap^r^ Tp^r^ colonies from each of the three independent targeted conservative experiments confirmed that in all instances, pRMH1010 had incorporated adjacent to IS*1216* in R388::IS*1216* via the targeted conservative cointegration mechanism.

### The *tnp26*, *tnp257*, and *tnp1216* genes are expressed equally in pUC19.

We considered the possibility that differences between the levels of *tnp257*, *tnp26*, and *tnp1216* transcription in the pUC19-based constructs are responsible for differences in the cointegration frequencies of IS*257* versus those of IS*26* and IS*1216*. The level of *tnp* expression was quantified relative to that of the constitutively expressed *bla*_TEM-1_ gene via reverse transcription-quantitative PCR (RT-qPCR) analysis of RNA isolated from constructs containing IS*26* (pRMH977), IS*257*R2 (pRMH1009), and IS*1216* (pRMH1010). No significant differences between *tnp* transcript levels were observed in three independent experiments (Table 4), indicating that the level of *tnp* expression is unlikely to be the cause of the lower cointegrate formation frequency of IS*257*.

**TABLE 4:** Expression of transposase genes in pUC19

Plasmid	IS (transposase gene)	Expression^*a*^
pRMH977	IS*26* (*tnp26*)	1
pRMH1008	IS*257*-3 (*tnp257-3*)	0.93 (0.88–1.13)
pRMH1009	IS*257*R2 (*tnp257R2*)	1.08 (0.79–1.33)
pRMH1010	IS*1216* (*tnp1216*)	0.84 (0.67–1.21)

a Expression relative to *tnp26* expression in pRMH977. *tnp* expression was determined in three independent experiments; the mean is reported and the range shown in parentheses.

## DISCUSSION

We predicted that the shared characteristics of members of the IS*26* family, namely, their related transposases and conserved TIRs, may indicate an ability to perform the copy-in and targeted conservative cointegration reactions (9). Here, we have experimentally shown for the first time that the IS*26* family members IS*257* and IS*1216* found in Gram-positive species form cointegrates by both the copy-in and conservative routes and hence share the dual-mechanistic cointegrate formation capability previously demonstrated only for IS*26*. However, the low frequency of cointegrate formation exhibited by IS*257* via both the copy-in and the targeted conservative route is surprising given the prevalence of IS*257* in many staphylococcal chromosomes and plasmids. This is an important step forward, as it extends this mechanism beyond the Gram-negative species in which IS*26* is found. Given the extent of the differences between Tnp26, Tnp257, and Tnp1216, it seems reasonable to conclude that all IS*26* family members can perform this reaction. Like IS*26*, the ability to function in two different modes has likely contributed to the success that IS*257* and IS*1216* have had in mobilizing antibiotic resistance genes and shaping the genomes of the species in which they reside.

IS*257* had previously been examined in more detail than IS*1216*, largely due to the association between IS*257* and determinants conferring resistance to antibiotics (aminoglycosides, bleomycin, mupirocin, tetracycline, trimethoprim, and virginiamycin), heavy metals (cadmium and mercury), antiseptics, and disinfectants in Staphylococcus aureus (1, 20). However, whether IS*257* is responsible for mobilizing these determinants is not always clear. There are numerous examples of small plasmids carrying resistance determinants [e.g., *tet*(K), *aadD*, and *erm*(C)] being integrated into a chromosome or other plasmids via IS*257*-mediated cointegration and generating an 8-bp TSD (14, 21). However, we could find only one example of the apparent movement of a transposon, Tn*4003*, resulting in the creation of an adjacent target site duplication (13). Further analysis of the now-extensive sequence data available will be needed to determine the true role of IS*257* in moving resistance genes in a transposon-like structure.

IS*1216* is associated with an increasing number of resistance genes in *Enterococcus* species (1, 18, 22), Clostridium perfringens (23), and S. aureus (24), including genes conferring resistance to penicillin, vancomycin, streptomycin, tetracycline-minocycline, gentamicin, kanamycin, and tobramycin, among others. However, like that of IS*257*, the role of IS*1216* in mobilizing these resistance genes is not always clear, and there are only a very limited number of cases where a TSD has been documented (16, 24).

Whereas IS*26* variants differ at only a few positions (19) and the IS*1216* isoforms (IS*1216*, IS*1216*V, and IS*1216*E) also differ at only a few positions, sharing at least 98.8% nucleotide identity to one another, the sequence divergence among the IS*257* isoforms is much greater (9). The degree of divergence of the IS*257* isoforms likely indicates a significantly longer evolutionary history of IS*257* in staphylococci, and it is possible that over time, IS*257* may have acquired mutations that have regulated the transposase activity to mitigate potentially deleterious effects in the host.

The findings reported here shed light on how IS*257* and IS*1216* form cointegrates and hence how they may mobilize antibiotic resistance genes. Clearly, further work on these key players in the modern resistance story in Gram-positive bacteria is warranted.

## MATERIALS AND METHODS

### Bacterial strains and media.

E. coli DH5α (*supE44* Δ*lacU169* [ϕ80 *lacZ*ΔM15] *hsdR17 recA1 endA1 gyrA96 thi-1 relA1)* was used to propagate plasmids. E. coli UB5201 (*pro met recA*, nalidixic acid resistance [Nx^r^]) was used as a donor in mating-out experiments, and E. coli UB1637 (*lys his trp lac recA* Sm^r^) was used as a recipient. MM383 [F^–^ *lacZ53 λ*^–^ *thyA36* IN(*rrnD-rrnE*)*1 rpsL151*(Sm^r^) *polA12*(ts) *rha-5 deoC2*] (25), a temperature-sensitive *polA* E. coli K-12 mutant, was used in temperature-sensitive cointegration assays, and an isogenic strain without the *polA* mutation, MM384 [F^–^ *lacZ53 λ*^–^ *thyA36* IN(*rrnD-rrnE*)*1 rpsL151*(Sm^r^) *rha-5 deoC2*), was included as a control. Antibiotics (Sigma) were added at the following concentrations to either Mueller-Hinton broth or Mueller-Hinton agar: ampicillin, 100 μg/ml; nalidixic acid, 25 μg/ml; streptomycin, 25 μg/ml; and trimethoprim, 25 μg/ml.

### Plasmid construction.

The plasmids used in this study are listed in Table 5. Gibson assembly (New England Biolabs, USA) was used to generate pRMH1008, pRMH1009, pRMH1010, R388::IS*257*-3, R388::IS*257*R2, and R388:IS*1216* using the primers listed in Table S1 in the supplemental material under standard manufacturer conditions. Inserts were cloned into the BamHI site of pUC19 or into the HindIII site of R388. pSK41 (26) DNA was used as the template for IS*257*-3 and IS*257*R2, and pJEG040 (27) DNA was used as the template for IS*1216*. The pUC19 universal primers were used to confirm the presence of the insert in pUC19, and primers RH2735 and RH2563 were used to confirm the presence of the insert in R388. PCR and routine sequencing of PCR products were performed as previously described (6) using primers listed in Table S1. Plasmid DNA was isolated by alkaline lysis as previously described (6).

**TABLE 5:** Plasmids used in this study

Plasmid	Description	Insert^*a*^	Resistance phenotype^*b*^	Reference
pRMH1008	IS*257*-3 in pUC19^*c*^	Bases 45556–100 from pSK41	Ap	This study
pRMH1009	IS*257*R2 in pUC19^*c*^	Bases 22809–23851 from pSK41	Ap	This study
pRMH1010	IS*1216* in pUC19^*c*^	Bases 25151–26035 from pJEG040	Ap	This study
R388	IncW plasmid		Su Tp	33
R388::IS*257*-3	IS*257-*3 in R388^*d*^	Bases 45556–100 from pSK41	Su Tp	This study
R388::IS*257*R2	IS*257*R2 in R388^*d*^	Bases 22809–23851 from pSK41	Su Tp	This study
R388::IS*1216*	IS*1216* in R388^*d*^	Bases 25151–26035 from pJEG040	Su Tp	This study

a pSK41, GenBank accession no. AF051917; pJEG040, GenBank accession number KX810025.

b Ap, ampicillin; Su, sulfamethoxazole; Tp, trimethoprim.

c The insert was cloned into the pUC19 BamHI site by Gibson assembly.

d The insert was cloned into the R388 HindIII site by Gibson assembly.

10.1128/mSphere.00811-19.1 TABLE S1 Primers used in this study. Download Table S1, DOCX file, 0.02 MB. Copyright © 2020 Harmer and Hall. 2020 Harmer and Hall This content is distributed under the terms of the Creative Commons Attribution 4.0 International license.

### Mating-out cointegration assays.

Donors for cointegration assays were generated via conjugation of either R388 (Su^r^ Tp^r^) or an R388 derivative containing the ISs of interest into E. coli UB5201 (*recA* mutant, Nx^r^) cells containing nonconjugative pUC19-derived plasmids containing IS*257*-3 (pRMH1008 Ap^r^), IS*257*R2 (pRMH1009 Ap^r^), or IS*1216* (pRMH1010 Ap^r^). Cointegrate formation was assessed by mating these strains with UB1637 (*recA* mutant, Sm^r^) and selecting for Ap^r^ Sm^r^ Tp^r^ colonies. pRMH977 (IS*26*) and R388::IS*26*, as previously tested (6, 7, 19), were included as a comparison. The transposition frequency was calculated as the number of Ap^r^ Sm^r^ Tp^r^ transconjugants (cointegrates) per Tp^r^ Sm^r^ transconjugant (R388 or R388 derivative). Targeted conservative cointegrate formation in R388::IS*257*-3, R388::IS*257*R2, or R388::IS*1216* was detected by PCR mapping across each IS into the R388 backbone using primers RH2563 and RH2735 (Table S1), flanking the R388 HindIII site, in combination with primers internal to IS*1216* (RH2738 and RH2739) or internal to IS*257*R2 (RH2736 and RH2737) (Table S1).

### Temperature-sensitive cointegration assay.

When cointegrate formation was below the limit of detection using the standard cointegration assay, a temperature-sensitive *polA* mutant strain, MM383, was used to detect cointegrate formation between the IS-containing pUC19-derived plasmid and the chromosome. ColE1-derived plasmids, such as pUC19, require DNA polymerase I (PolI) to initiate replication (28), and MM383 is PolI defective at 42°C, resulting in the loss of a pUC19-derived plasmid when grown at the nonpermissive temperature unless it is incorporated into the chromosome, e.g., via IS-mediated cointegrate formation.

pUC19 (Ap^r^), pRMH1008 (pUC19::IS*257-3* Ap^r^), or pRMH1009 (pUC19::IS*257*R2 Ap^r^) was transformed into MM383 (Sm^r^) by electroporation as described previously (8). The resulting Ap^r^ Sm^r^ transformant was purified and grown at 32°C overnight (∼16 h) in 5 ml LB supplemented with Ap and Sm. One milliliter of overnight culture was inoculated into 100 ml of prewarmed LB without ampicillin selection for the plasmid and grown at 42°C for 24 h. At the end of the growth period, the culture was serially diluted in 0.9% (wt/vol) saline and plated onto LB agar supplemented with Sm to select for all MM383 cells or supplemented with Ap and Sm to select for MM383 with the plasmid integrated and incubated overnight at 32°C. Resistance to Ap was indicative of cointegrate formation between the pUC19-derived construct and the chromosome. Fifteen Ap^r^ Sm^r^ colonies were subjected to a second round of growth at the nonpermissive temperature to ensure that Ap^r^ was stably maintained, verifying that Ap^r^ was indicative of cointegrate formation, rather than the presence of residual free plasmid.

### Inverse PCR and sequencing.

Inverse PCR (29) and sequencing were used to map the junctions of the pUC plasmid with the chromosome or R388 formed via the untargeted copy-in reaction. Whole-cell DNA was prepared by alkaline lysis (30). NEBcutter version 2.0 (http://nc2.neb.com/NEBcutter2) (31) was used to identify restriction enzymes that would digest the backbone frequently but would not digest the IS or pUC19-derived fragment (i.e., the internal cointegrate fragment). Two micrograms of whole-cell DNA was digested with 5 units of BtgI (for cointegrates formed by IS*257*) or 5 units of BsmI (for cointegrates formed by IS*1216*) at 37°C for 2 h. Ten nanograms of digested DNA was added to a 10-μl ligation reaction mixture (200 U T4 DNA ligase, 1.0 μl T4 DNA ligase buffer) and incubated at room temperature for 8 h. Three microliters of the ligation reaction mixture was used as the template in an inverse PCR performed using primers internal to the ISs: primers RH2736 and RH2737 (Table S1) for cointegrates formed by pRMH1008 and pRMH1009 and primers RH2738 and RH2739 (Table S1) for cointegrates formed by pRMH1010. To determine the cointegrate boundaries, products from the inverse PCR were visualized on a 1% Tris-acetate-EDTA (TAE) gel, followed by gel extraction and sequencing with primers RH2736/RH2737 or RH2738/RH2739 as described previously (6).

### qRT-PCR.

Quantitative real-time PCR was performed as described previously (32), using primers (Table S1) RH1464 and RH1465 to detect the expression of *tnp26*, RH2740 and RH2741 to detect *tnp257R2* or *tnp257-3*, and RH2742 and RH2743 to detect *tnp1216*. Constitutively expressed *bla*_TEM-1_ from the plasmid backbone was used as an endogenous control (primers RH1466 and RH1467). Real-time PCR was performed in triplicate on independent biological-replicate samples.
