# Supplementary material for: IS26 Family Members IS257 and IS1216 Also Form Cointegrates by Copy-In and Targeted Conservative Routes
Source: mSphere. 2020 Jan 8;5(1):e00811-19. doi: 10.1128/mSphere.00811-19 (PMC6952201; doi:10.1128/mSphere.00811-19)
Supplement: TABLE S1 [file mSphere.00811-19-st001.docx]

Table S1. Primers used in this study.

| Primer | Target | Sequence (5′ – 3′)^a^ |
| --- | --- | --- |
| *Gibson cloning into pUC19 BamHI site* | |  |
| RH2712 | IS*257*-3 in pSK41 | **ATTCGAGCTCGGTACCCGGG**CAAAGTCCTCATTCATTTC |
| RH2713 | IS*257*-3 in pSK41 | **CCTGCAGGTCGACTCTAGAG**CCTTCGTGATCAAAATCTAAATTC |
| RH2720 | IS*257*R2 in pSK41 | **ATTCGAGCTCGGTACCCGGG**ATTTTTCCGTTCCCAATTC |
| RH2721 | IS257R2 in pSK41 | **CCTGCAGGTCGACTCTAGAG**CGCACAAGAAAAAGATTTC |
| RH2727 | IS*1216* in pJEG040 | **ATTCGAGCTCGGTACCCGGG**CGAGTGATTCGAGTTAAGTC |
| RH2728 | IS*1216* in pJEG040 | **CCTGCAGGTCGACTCTAGAG**AGAACCGGAAAGCAGAATG |
|  |  |  |
| *Gibson cloning into R388 HindIII site* | |  |
| RH2729 | IS*257*R2 in pSK41 | **AGCGAGGGCTTTACTA**GTGTCATCAAGGTTTAATTTTTG |
| RH2730 | IS*257*R2 IN PSK41 | **AAGCCCCACGCATCA**CACAAACAGTAAAGATGGTTC |
| RH2731 | IS*257*-3 in pSK41 | **AGCGAGGGCTTTACTA**CAAAGTCCTCATTCATTTC |
| RH2732 | IS*257*-3 in pSK41 | **AAGCCCCACGCATCA**CCTTCGTGATCAAAATCTAAATTC |
| RH2733 | IS*1216* in pJEG040 | **AGCGAGGGCTTTACTA**CGAGTGATTCGAGTTAAGTC |
| RH2734 | IS*1216* in pJEG040 | **AAGCCCCACGCATCA**CACAAACAGTAAAGATGGTTC |
|  |  |  |
| *Inverse PCR primers* | |  |
| RH2736 | IS*257*R2 | GTTCATACAGAAGACTCCTTTTTGTT |
| RH2737 | IS*257*R2 | CCATGCCACGAAATTAGCAT |
| RH2738 | IS*1216* | TCCTGCTGAAATTGCTTTCC |
| RH2739 | IS*1216* | GGGAATCCCAGCTTAAATCA |
|  |  |  |
| *Screening/Mapping primers* | |  |
| RH2735 | Upstream of R388 HindIII site | GCCCTACACAAATTGGGAGA |
| RH2563 | Downstream of R388 HindIII site | GCAATTATGAGCCCCATACC |
|  |  |  |
| Real-time PCR primers | |  |
| RH1466 | *bla*TEM-1 in pUC19 | AAGCCATACCAAACGACGAG |
| RH1467 | *bla*TEM-1 in pUC19 | TTGCCGGGAAGCTAGAGTAA |
| RH1464 | IS*26* | ACCTTTGATGGTGGCGTAAG |
| RH1465 | IS*26* | TACCGGAACAACGTGATTGA |
| RH2740 | IS*257*R2 or IS*257-3* | AAGGGAACGTGGTGTAAACG |
| RH2741 | IS*257*R2 or IS*257-3* | TCGCGCGATACAGATAACAC |
| RH2742 | IS*1216* | TTGAACAAGACCATCGTCCA |
| RH2743 | IS*1216* | TACAGACCGAAAACCCGAAG |

a Sequence in bold denotes the pUC19- or R388-specific portion of a Gibson primer, and the underlined sequence denotes the insert-specific portion.
